# Removal of mercury(II) from aqueous solution by partially reduced graphene oxide

**DOI:** 10.1038/s41598-022-10259-z

**Published:** 2022-04-19

**Authors:** Talia Tene, Fabian Arias Arias, Marco Guevara, Adriana Nuñez, Luis Villamagua, Carlos Tapia, Michele Pisarra, F. Javier Torres, Lorenzo S. Caputi, Cristian Vacacela Gomez

**Affiliations:** 1 Departamento de Química, Universidad Técnica Particular de Loja, 110160 Loja, Ecuador; 2 Facultad de Ciencias, Escuela Superior Politécnica de Chimborazo (ESPOCH), 060155 Riobamba, Ecuador; 3 ITECA-Instituto de Tecnologías y Ciencias Avanzadas, Villarroel y Larrea, 060104 Riobamba, Ecuador; 4 UNICARIBE Research Center, University of Calabria, 87036 Rende, CS Italy; 5 Departamento de Ciencias de la Computación y Electrónica, Universidad Técnica Particular de Loja, 110160 Loja, Ecuador; 6 INFN, sezione LNF, Gruppo collegato di Cosenza, Cubo 31C, 87036 Rende, CS Italy; 7 Grupo de Química Computacional y Teórica (QCT-UR), Facultad de Ciencias Naturales, Universidad del Rosario, Bogotá, Colombia; 8 Grupo de Química Computacional y Teórica (QCT-USFQ), Insituto de Simulación Computacional (ISC-USFQ), Departamento de Ingeniería Química, Universidad San Francisco de Quito, Diego de Robles y Vía Interoceánica, Quito, Ecuador; 9 Surface Nanoscience Group, Department of Physics, University of Calabria, Via P. Bucci, Cubo 33C, 87036 Rende, Italy

**Keywords:** Environmental chemistry, Environmental impact

## Abstract

Mercury (Hg(II)) has been classified as a pollutant and its removal from aqueous sources is considered a priority for public health as well as ecosystem protection policies. Oxidized graphenes have attracted vast interest in water purification and wastewater treatment. In this report, a partially reduced graphene oxide is proposed as a pristine adsorbent material for Hg(II) removal. The proposed material exhibits a high saturation Hg(II) uptake capacity of 110.21 mg g^−1^, and can effectively reduce the Hg(II) concentration from 150 mg L^−1^ to concentrations smaller than 40 mg L^−1^, with an efficiency of about 75% within 20 min. The adsorption of Hg(II) on reduced graphene oxide shows a mixed physisorption–chemisorption process. Density functional theory calculations confirm that Hg atom adsorbs preferentially on clean zones rather than locations containing oxygen functional groups. The present work, therefore, presents new findings for Hg(II) adsorbent materials based on partially reduced graphene oxide, providing a new perspective for removing Hg(II).

## Introduction

Hg(II) pollution-which can cause significant neurodevelopmental risk to fetuses, newborns, and children- has long been considered a threat to public health and the environment^1^. The release of Hg(II) into surface water occurs mainly through discharge from industrial processes such as automobile manufacturing^2^, oil refinery^3^, battery facilities^4^, military wastes^5^, and fossil fuel combustion^6^. To decrease the level of exposure of human beings to Hg(II), a global agreement has been reached, spurring the research to remove and recover Hg(II) ions from industry wastewater^7^. In this regard, the world health organization (WHO) has determined that the maximum allowable concentration of Hg(II) in drinking water and wastewater discharge is 1 μg/L and 5 μg/L, respectively^8^.

Numerous methods and technologies have been developed for Hg(II) removal, including precipitation, coagulation, membrane filtration, solvent extraction, photocatalysis, ion exchange, and adsorption^9–11^. Among all these techniques, adsorption holds great promise due to the simplicity, high adsorption rate, non-secondary treatment step, and relatively low-cost technology^12–14^. A variety of conventional adsorbents have been proposed for removing Hg(II) from contaminated waters, namely, activated carbons^15^, zeolites^16^, resins^17^, mesoporous silica^18^, mesoporous carbons^19^, and clays^20^. However, these adsorbents have presented low adsorption capacity and weak binding affinity for Hg(II) ions. On the other hand, metal–organic frameworks^21^ or functionalized organic polymers^22^ are also reported as effective sorbents for Hg(II) removal due to their high surface areas. Nevertheless, these new types of adsorbents usually show instability in water or aqueous solutions, reducing the effectiveness of the adsorption method to purify water^23^.

Nowadays, existing Hg(II) adsorbent materials still face sorts of challenges such as low surface area, low efficiency, and complicated post-processing, making their practical use for water treatment less likely^24^. Because of the weaknesses and handicaps associated with existing adsorbents, it is important to develop new types of materials for highly effective and highly efficient removal of Hg(II) from aqueous solutions. To tackle the aforementioned limitations, carbon nanomaterials with a higher surface area have been proposed, such as carbon nanotubes^25^, graphene oxide (GO)^26^, and reduced graphene oxide (rGO)^27^. Among them, rGO has attracted increased attention as an efficient adsorbent of dyes^28^ and heavy metal ions^29^ due to its hydrophilic properties, biocompatibility, and cost-effective preparation method^30^.

Both GO and rGO are characterized by the presence of different oxygen functional groups (hydroxyl, epoxide, carbonyl, and carboxylic groups^31,32^) that allow covalent modifications with strong chelating groups, which in turn, present a high affinity to coordinate with metal ions^33^. Although GO and rGO are interesting adsorbent materials, GO is characterized by a higher hydrophilic character that could interfere with the extraction of heavy metals. On the other hand, GO or rGO functionalized with amines or thiols have been utilized as new adsorbents for the removal of heavy metals from water with a remarkable high selectivity and capacity for Hg(II) ions^34^. However, the involved process: oxidation, reduction, preparation of extra-functionalized material, and final purification, are a bottleneck when using GO or rGO for large-scale water treatment technologies. We have recently reported on a scalable and eco-friendly protocol to prepare rGO with promising environmental applications^35^.

Here we show the Hg(II) removal using a green-prepared rGO which exhibits an interesting Hg(II) saturation of 110.21 mg g^−1^ (at 298 K) and can effectively decrease the Hg(II) concentration of 150 mg L^−1^ at the low level of less than 40 mg L^−1^. Furthermore, rGO can efficiently remove $\sim 75$% Hg(II) in 20 min, outperforming conventional Hg(II) removal adsorbents (discussed below). While rGO cannot be considered as a universal solution to treat the different existing pollutants, for an illustrative comparison, the results of the adsorption of methylene blue (MB)^36^ are also presented since it is a cationic pollutant. The present work describes in detail the adsorption process of Hg(II) on as-made rGO (never reported) and shows several advantages such as green adsorbent synthesis, easy adsorption, absence of toxic gases evolution in the oxidation–reduction process and, most importantly, non-extra functionalization. We complete the study exploring the Hg-rGO interaction mechanism by means of gas-phase and periodic state-of-the-art density functional theory calculations. Our results are expected to be of immediate help in the application of rGO in water treatment technologies.

## Results

### Synthesis and characterization

GO was transformed into rGO using citric acid (CA) as a reducing agent and following our eco-friendly protocol reported in Ref.^35^ (briefly described in “Methods”). The successful preparation of rGO (without further functionalization) was confirmed by transmission electron microscopy (TEM), scanning electron microscopy (SEM), energy-dispersive X-ray spectroscopy (EDS), UV–Vis spectroscopy (UV–Vis), Raman, and X-ray diffraction (XRD) analyses.

**Figure 1:**
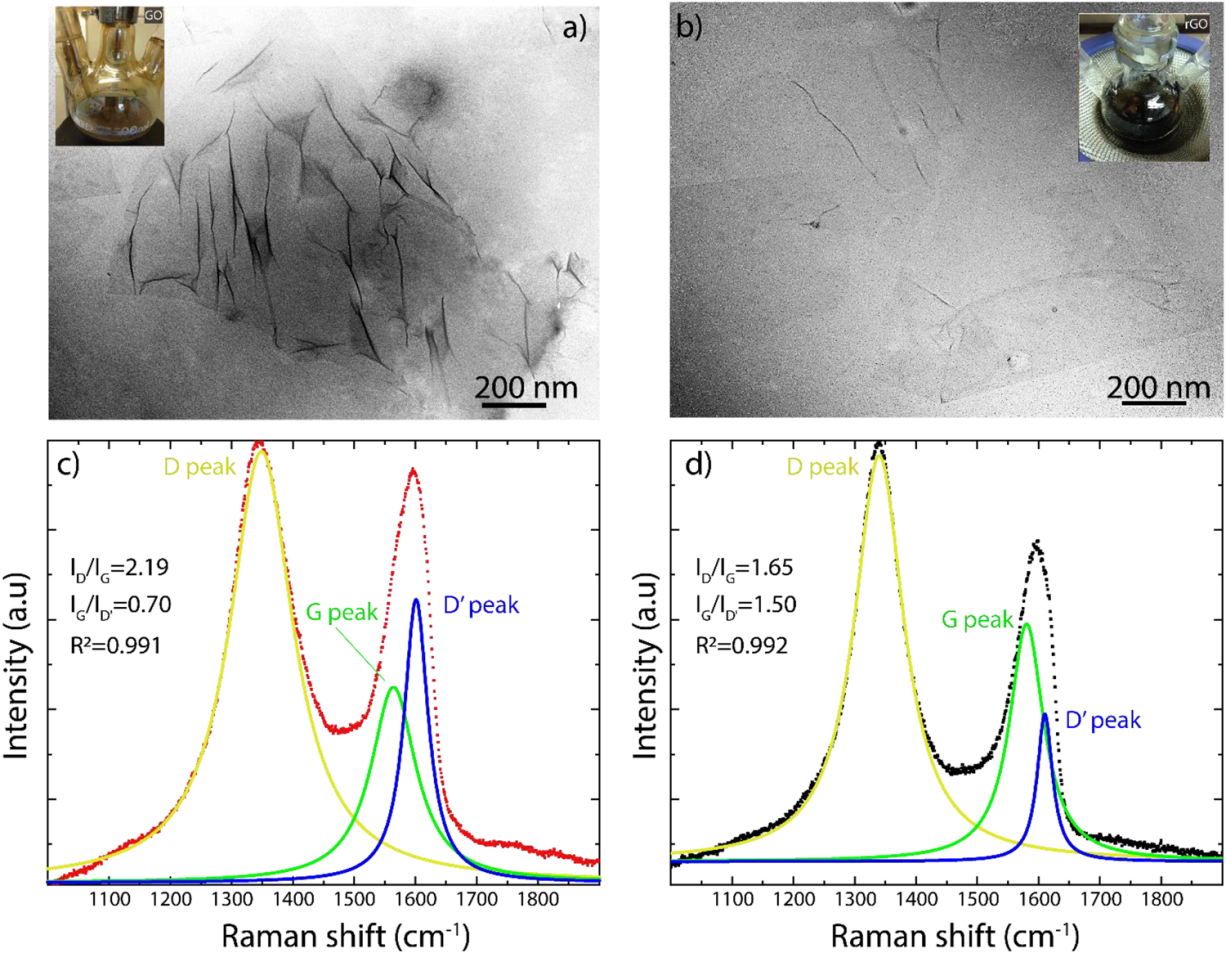
TEM and Raman studies. TEM images of (**a**) GO and (**b**) rGO. Raman spectra of (**c**) GO and (**d**) rGO. Inset images show the optical transformation of GO (yellowish suspension) into rGO (blackish suspension).

To begin, TEM images of GO and rGO are shown in Fig. 1. GO appears as a semi-transparent and thin nanosheets with various wrinkles and folds on the surface and edges (Fig. 1a). The wrinkled structure is associated with surface defects due to the deviation from sp^2^ to sp^3^ character as a consequence of a high density of oxygen functional groups^32,37^. After the reduction process, rGO is characterized by well-defined and impurity-free nanosheets with slightly wrinkled regions (Fig. 1b), suggesting recovery of sp^2^ hybridization by the removal of oxygen functional groups, explicitly, the diminution of the degree of oxidation^32^. Inset images evidence the optical transformation of GO (yellowish suspension) into rGO (blackish suspension).

As TEM, SEM micrographs show similar features. GO exhibits a randomly aggregated flaky sheet with wrinkles and folds (Fig. S1a). Instead, rGO reveals a highly distorted surface morphology with micropore formation (Fig. S1c). Elemental analysis of the carbon and oxygen content in GO (Fig. S1b) and rGO (Fig. S1d) was measured by EDS. The oxygen content decreased by 26.2% after the reduction process, confirming the removal of the oxygen functional groups.

UV–Vis spectra demonstrated that GO has the main absorption peak at 230 nm (green curve) and a shoulder peak at 329 nm (yellow curve) (Fig. S2a), which are related to the $\pi {-}{\pi }^{*}$ transitions (C–C bonds) and $n{-}{\pi }^{*}$ transitions (C=O bonds), respectively^35–37^. The latter peak indicates the presence of C–O functional groups. We emphasize that two important features must be observed in the transformation of GO into rGO: (i) a redshift of the main absorbance peak and (ii) a loss of the shoulder peak. The as-made rGO only meets the first requirement, the first absorbance peak shifts at 261 nm (green curve) whereas the second one remains at 324 nm (yellow curve) (Fig. S2b), demonstrating the presence of a partially reduced material.

This partial reduction was further confirmed by Raman spectroscopy. GO is characterized by three prominent peaks in the window of 1000–2000 cm^−1^: the D peak (yellow curve), G peak (green curve), and D′ peak (blue curve) (Fig. 1c). The G peak is related to the in-plane motion of sp^2^-hybridized carbon atoms in the graphene/graphite lattice, while the D and D′ peaks are related to the presence of basal/edge defects and the change from sp^2^ to sp^3^ hybridization^38^. A decrease of the D’ peak intensity is a direct indication of GO reduction^37^. Although a significant decrease in the intensity of the D' peak is noticed in rGO (Fig. 1d), this peak is still observed, confirming the presence of a partially reduced material.

The crystallinity changes from GO to rGO were revealed by XRD analysis (Fig. S3a). Raw graphite is characterized by an intense crystalline peak at $2\theta =26.73^\circ$ with a lattice spacing of 0.334 nm, which corresponds to the (002) diffraction peak^39^. In GO, this peak is found at $2\theta =10.93^\circ$ with a lattice spacing of 0.81 nm, indicating the oxidation of graphite. The increased interlayer spacing is attributed to the intercalation of water molecules and oxygen functional groups^40^. As well, the very low width of this peak testifies an ordered stacking along the out-of-plane axis. After the reduction process, this peak becomes broader due to the partial breakdown of the long-range order^40^, and it shifts towards higher angles, $2\theta >22^\circ$, showing a decrease in the lattice spacing ($\sim$ 0.39 nm).

To determine the thermal stability of as-made materials and the effect on the presence of oxygen functional groups, we carried out thermogravimetric analyses (TGA) on GO and rGO (Fig. S3b). In GO, the weight loss before 100 °C is ascribed to the loss of water molecules. The significant weight loss in the region of $$200{-}300 $$ °C is attributed to the pyrolysis of unstable molecules (such as CO, CO_2_, and H_2_O)^35^. In the region of $$300{-}600$$ °C, the weight loss is due to the removal of stable oxygen functional groups. Instead, rGO shows relative thermal stability but the observed TGA curve follows a similar trend as GO, suggesting a comparable presence of oxygen functional groups^35^.

All these facts demonstrate that the reduction of GO has taken place using CA as a reducing agent, i.e., we attained a green prepared rGO but it is partially reduced material, which will be used for the adsorption of Hg(II).

### Hg(II) sorption kinetics

To evaluate the effectiveness of rGO for removing Hg(II) from water, as-made rGO was placed in a dilute aqueous solution of HgO (pH $=6.4$) with a Hg(II) concentration of 150 mg L^−1^. The adsorption capacity (${q}_{t}$) was determined by the following expression:

1 $${q}_{t}=\frac{\left({C}_{0}-{C}_{t}\right) V}{W}$$

where ${C}_{0}$ is the initial Hg(II) concentration (mg L^−1^) and ${C}_{t}$ is the Hg(II) concentration (mg L^−1^) at the time, $t$. $V$ represents the volume of the solution (L), and $W$ is the adsorbent mass (g). At the equilibrium, the equilibrium concentration (${C}_{e}$) and equilibrium adsorption capacity (${q}_{e}$) is ${C}_{e}={C}_{t}$ and ${q}_{e}={q}_{t}$, respectively.

The removal effectiveness ($RE\%$) of the adsorbent material is defined as:

2 $$RE\% =\frac{\left({C}_{0}-{C}_{e}\right) }{{C}_{0}}\times 100$$

**Figure 2:**
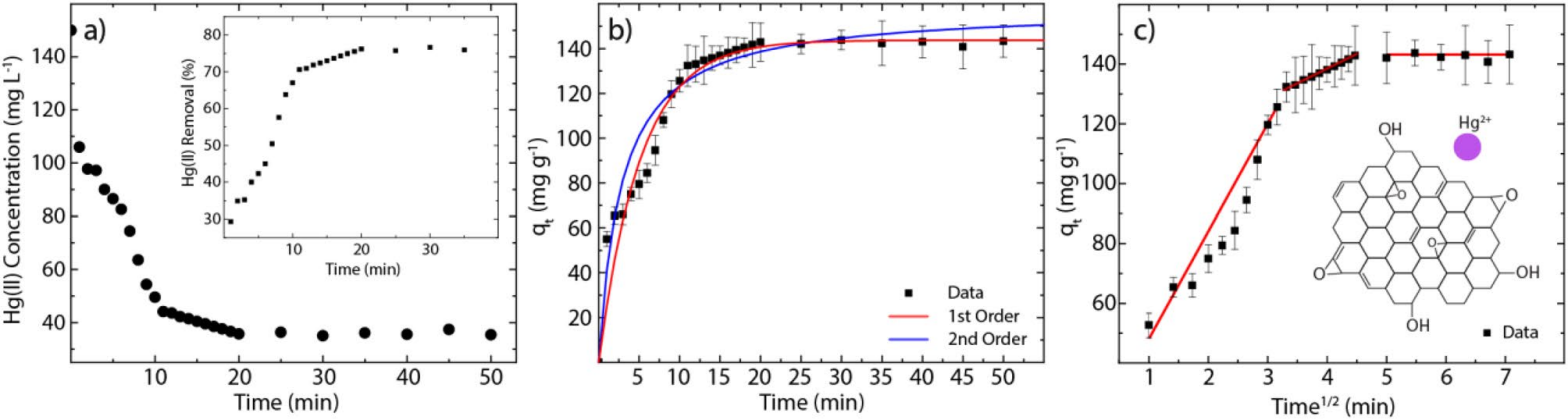
Kinetics investigation and particle diffusion. (**a**) Hg(II) adsorption kinetics of rGO under the Hg(II) initial concentration of 150 mg L^−1^. Inset shows the removal percentage. (**b**) The pseudo-first and pseudo-second-order kinetic plot for the adsorption (Hg(II) concentration 150 mg L^−1^). (**c**) Intraparticle diffusion plot showing three regions of linearity (Hg(II) concentration 150 mg L^−1^).

As shown in Fig. 2a, rGO can rapidly capture Hg(II) ions after 20 min. The residual Hg(II) concentration in the solution was less than 40 mg L^−1^, which means that about 75% of Hg(II) was removed by rGO (inset Figure). It is worth mentioning, the residual Hg(II) concentration in the solution treated with rGO is 2.4 times lower than that found in the solution treated with sulfydryl-functionalized graphene oxide (s-GO) at the same time^41^. This value is also lower than that found in the solution treated with polyamine modified reduced graphene oxide rich in amino groups (HT-rGO-N)^42^ or graphene oxide nanoribbons (GONRs)^43^. These results highlight the effectiveness of as-made rGO for removing Hg(II) from aqueous solutions compared with some graphene-based benchmark sorbents.

To further highlight the feasibility of rGO, Fig. S4 shows the adsorption of Hg(II) onto as-made GO at 298 K. While GO can capture Hg(II) ions after 10 min, the residual Hg(II) concentration in the solution is about 107 mg L^−1^ (Fig. S4a), which means that just 28% of the Hg(II) was removed by GO (Fig. S4b). Thus, the effectiveness of rGO for the Hg(II) removal could be attributed to two important facts: (i) the presence of oxygen functional groups as well as (ii) the oxygen-free zones recovered after the reduction process. These facts will be analyzed in detail in the remainder of the article.

Now we focus on the adsorption mechanism of Hg(II) onto rGO at 298 K. For an illustrative comparison, the adsorption of MB on as-made rGO is also reported (Supplementary Material)^36^. The adsorption kinetic curve as a function of time is reported in Fig. 2b. As observed, the adsorption equilibrium time for the Hg(II) removal was reached after 20 min. However, it takes about 30 min to remove the MB molecules (Fig. S5a). The adsorption kinetic parameters were determined by the pseudo-first- and pseudo-second-order models. The first model assumes that the rate of change of solute uptake with time is directly proportional to the difference in saturation concentration and the amount of solid uptake with time^44^. The involved parameters can be estimated as follow:

3 $$\mathrm{log}({q}_{e}-{q}_{t}) =\mathrm{log}{q}_{e}-\frac{{k}_{1}}{2.303}t$$

where ${k}_{1}$ represents the pseudo-first-order rate constant.

The second model adopts that the rate-limiting step is chemical sorption or chemisorption and predicts the behavior over the whole range of adsorption^44^. The experimental data can be fitted using the following equation:

4 $$\frac{t}{{q}_{t}} =\frac{1}{{k}_{2}{q}_{e}^{2}}+\frac{1}{{q}_{e}}t$$

where ${k}_{2}$ denotes the pseudo-second-order kinetic rate constant. The estimated parameters of the adsorption kinetics are summarized in Table S1.

From the pseudo-first-order model (red curve), the calculated ${q}_{e(cal)}$ value (${q}_{e(cal)}=143.71$ mg g^−1^) is very close to the experimental value (${q}_{e}=142.26$ mg g^−1^). In contrast, the pseudo-second-order model (blue curve) slightly overestimates the equilibrium adsorption capacity (${q}_{e(cal)}=151.32$ mg g^−1^). When comparing the values of SSE and R^2^, the adsorption kinetics of Hg(II) on rGO is more in line with the pseudo-first-order model. Nevertheless, the pseudo-second-order model cannot be completely ruled out (R^2^ = 0.931) since it suggests that the adsorption process of Hg(II) onto rGO could be controlled by physisorption or chemisorption (discussed below).

### Hg(II) ion diffusion

The diffusion process of metal ions into porous solid materials can be studied by the intraparticle diffusion (IPD) model, which mainly involves various steps characterized by different rates. The most widely used IPD equation is given by the following expression^45^:

5 $${q}_{t} ={k}_{p}{t}^{0.5}+C$$

where ${k}_{p}$ is the intraparticle diffusion rate constant (g mg^−1^ min) and $C$ is the intercept of the plot, which reflects the boundary layer effect or surface adsorption^44^. The estimated parameters of the IPD model are summarized in Table S2. As is well known, the larger the intercept value, the greater the contribution of the surface adsorption in the rate-limiting step^46^. Then the observed value of surface adsorption ($C=44.28$ mg g^−1^) suggests that a greater amount of surface adsorption occurred, leading to a decrease in the rate of diffusion of Hg(II) ions from the external surface to the internal structure. This fact is exposed by analyzing the linearized plot of the diffusion of Hg(II) ions into the rGO structure (Fig. 2c).

Three regions are observed: (i) the initial region (faster stage) is related to the movement of Hg(II) ions from solution to the rGO surface, (ii) the second region is related to the gradual diffusion of Hg(II) ions into the larger pores of rGO structure, and (iii) the final stage involves a very slow diffusion of Hg(II) ions from larger pores to smaller ones. In contrast, only the first and second regions are observed in the diffusion of MB into rGO (Fig. S5b). This fact could be attributed to the different adsorption mechanisms involved and to the size of the MB molecule, which play an important role in the diffusion from larger to smaller pores.

To further explore the mentioned regions in the Hg-rGO system, the initial adsorption factor (${R}_{i}$) should be calculated as:

6 $${R}_{i} =\frac{{q}_{ref}-C}{{q}_{ref}}$$

where $C$ is the ratio of the initial adsorption amount and ${q}_{ref}$ is the final adsorption amount at the longest time. The calculated ${R}_{i} \sim 0.49$ indicates a limit between the strong initial adsorption and intermediate initial adsorption^46^, which means that the adsorption of Hg(II) ions would occur on the surface of rGO. For high surface materials, the adsorption kinetics has a strong degree of surface adsorption, that is, most of the adsorption occurs on the adsorbent surface^44^. This fact is not fully observed in rGO. A feasible explanation can be the partially recovered surface area (sp^2^ character) of rGO as a consequence of a partially reduced material.

### Hg(II) sorption isotherms

To assess the Hg(II) uptake capacity of rGO, which is an important aspect and metric, adsorption isotherm for Hg(II) removal from the water was collected with a span of 20 min (the equilibrium time). The experimental data can be fitted using the Langmuir and Freundlich models, by the following expressions:

7 $${q}_{e} =\frac{{q}_{m}{K}_{L}{C}_{e}}{1+{K}_{L}{C}_{e}}$$

8 $$\mathrm{log}{q}_{e}=\mathrm{log}{K}_{F}+\frac{1}{n}\mathrm{log}{C}_{e}$$

**Figure 3:**
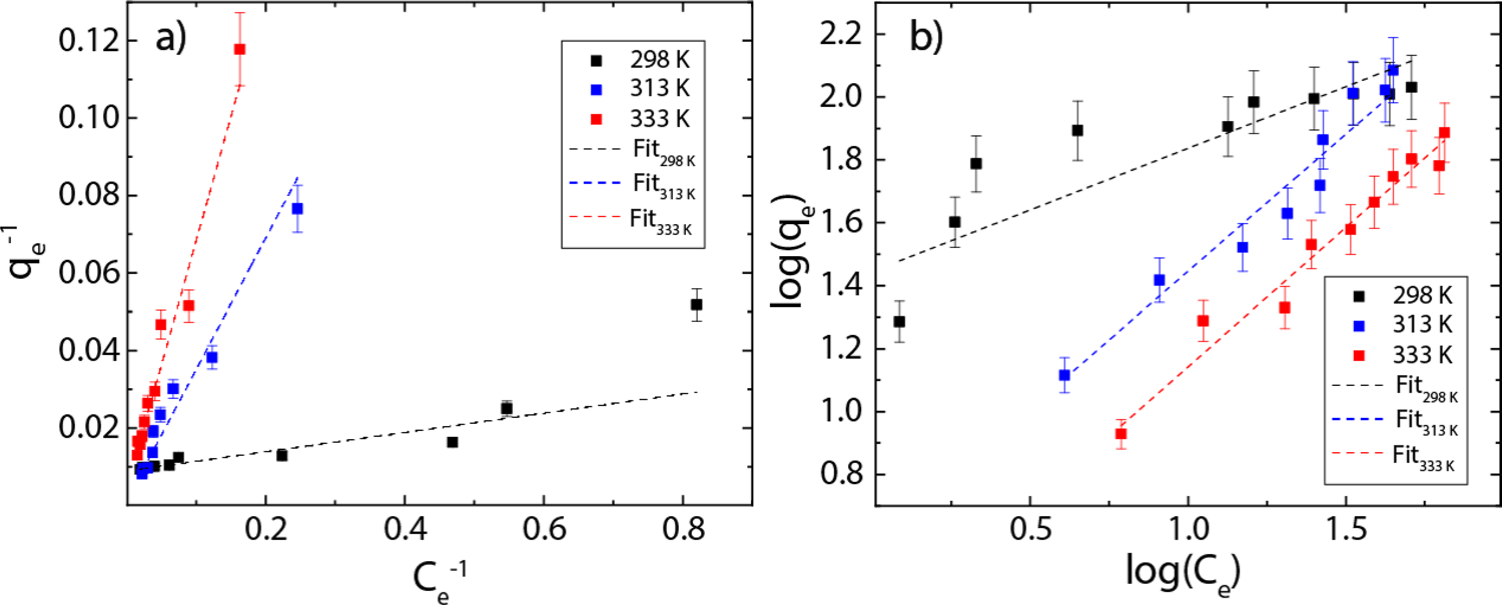
Hg(II) adsorption isotherms for rGO. (**a**) Langmuir linear model (**b**) Freundlich linear model.

Equation (7) is the Langmuir linear model where ${q}_{m}$ represents the maximum adsorption capacity (mg g^−1^) and ${K}_{L}$ is the Langmuir constant (L g^−1^). Equation (8) is the Freundlich linear model where ${K}_{F}$ is the adsorption capacity and $n$ is the heterogeneity of adsorbent material. The corresponding results and estimated parameters are presented in Fig. 3 and Table S3, respectively.

Based on the high correlation R^2^ value, it can be seen that the experimental data is slightly more in line with the Langmuir model regardless of the temperature (Table S3). Although temperature does not dramatically change the chemical composition of rGO ($<100^\circ{\rm C}$) as shown by TGA results (Fig. S3b), it seems to be an important parameter in the adsorption of Hg(II) ions. By increasing the temperature from 298 to 333 K, the maximum adsorption capacity (${q}_{m}$) significantly increases from 110.21 to 255.04 mg g^−1^ (Fig. 3a, Table S3). Instead, an inverse relationship is observed for MB, the ${q}_{m}$ values decrease from 121.95 to 107.53 mg g^−1^ with increasing the temperature (Fig. S6a).

The higher ${q}_{m}$ values observed for the Hg(II) removal at temperatures above 298 K, can be attributed to the fact that these values are estimated depending on data trend. Keeping this in mind, the effectiveness of adsorbent material should be based on the percent of Hg(II) removal^13^. Beyond the effect of temperature, the estimated ${q}_{m}$ value at 298 K (${q}_{m}=110.21$ mg g^−1^) is higher than those of recent studies (see Table 1), suggesting that as-made rGO is a promising material compared to functionalized/decorated GO, rGO, or even more complex carbon-containing structures (Fe_1 −x_S NP/C microspheres^47^).

**Table 1:** Comparative maximum adsorption capacity, time, and pH of several adsorbents for the Hg(II) removal.

Adsorbents	Adsorption capacity (mg g^−1^)	Time (min)	pH	Ref
Fe_1−x_S NP/C microspheres	104	250	6.5	^47^
GONR (Hg and As)	33.02	12	6.0	^43^
S-GO	3490	240	1–12	^41^
GO-TSC	231	30	3.5	^48^
S-doped g-C_3_N_4_/LGO	46	120	5.0	^49^
GSH-NiFe_2_O_4_/GO	272.94	90	6.0	^50^
HT-rGO-N	75.8	10	5–9	^42^
This work	110.21	20	6.4	

In addition to high maximum adsorption capacity, rGO is also more efficient in adsorption time (20 min, pH $=6.4$). Particularly, S-GO seems to be more profitable for the Hg(II) removal since, obviously, the presence of sulfur improves the affinity and specificity for Hg(II) ions. However, its adsorption equilibrium time (240 min) is 12 times higher than the present study.

By analyzing the Freundlich model (Table S3), the $n$ values (0.48–1.14) found at different temperatures (298–333 K) indicate that the adsorbent heterogeneity is minimal and tends to be non-homogeneous as temperature increases^36,44^. In point of fact, values of $n$ very close to zero indicate strong surface heterogeneity. It is also evident (Fig. 3) that the Langmuir and Freundlich models become equivalent at 313 and 333 K because $n\approx 1$^44^ (Table S3). The affinity of rGO for Hg(II) ions can be elucidated by the ${K}_{L}$ parameter, where the respective values were found to be $>0.1$, suggesting a good affinity.

### Effect of initial concentration and pH

**Figure 4:**
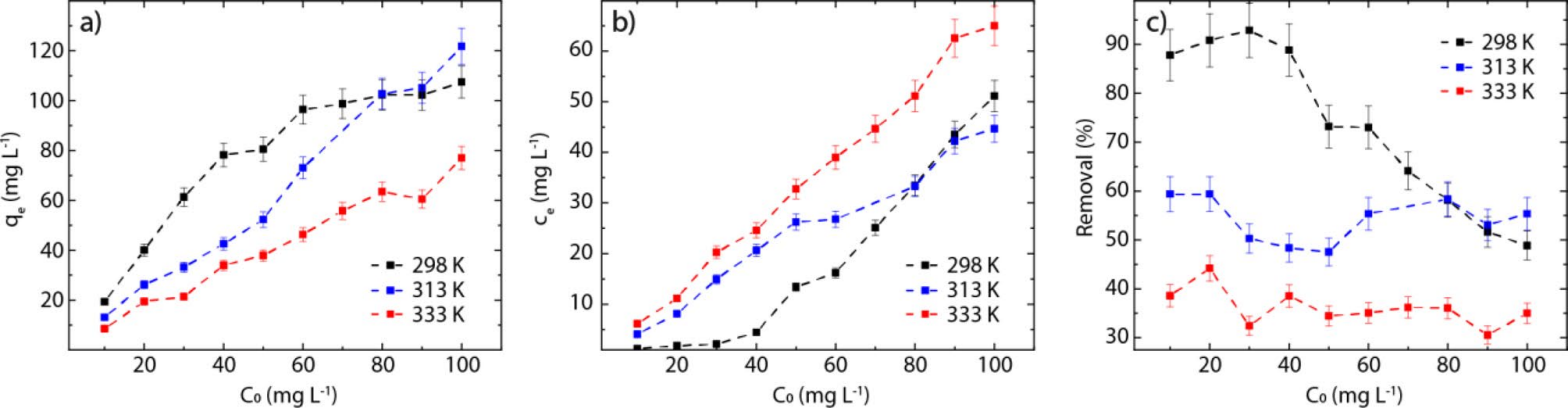
Effect of the initial concentration. (**a**) The adsorption capacity of rGO as a function of the initial Hg(II) concentration, (**b**) ${C}_{e}$ as a function of ${C}_{0}$, and (**c**) removal percentage as a function of ${C}_{0}$, considering three different temperatures (298, 313, 333 K).

The adsorption capacity (${q}_{e}$) of rGO increases linearly with the initial concentrations of Hg(II) in solution (${C}_{0}$), particularly, in the range from 10 to 50 mg L^−1^ at 298 K and from 10 to 90 mg L^−1^ at 313 and 333 K. At higher concentrations a deviation from linearity does occur (Fig. 4a). This suggests that rGO has a limited number of adsorbent sites, which is fixed by its quantity and by the experimental conditions (e.g., the temperature, pH, and solution volume/adsorbent mass ratio). To clarify this idea, at the beginning of the adsorption process, rGO is characterized by a vast number of active sites, increasing the ${q}_{e}$ value as long as free active sites are available. When all the active sites are involved, saturation is reached, and therefore the maximum adsorbent capacity (${q}_{m}$). The latter statement is noticeable at 298 K (black points).

The equilibrium concentration of Hg(II) increases with ${C}_{0}$ (Fig. 4b). It is worth noting that at low initial concentrations ($<10$ mg L^−1^), the equilibrium concentration at 298 and 313 K falls in the ppb range. On the other hand, at 313 and 333 K, the adsorption effectiveness of rGO (defined as the percentage of the Hg(II) removal from water, Fig. 4c) is almost independent of ${C}_{0}$, assuming an average value of 54.12% and 39.10%, respectively. A clear dependence on ${C}_{0}$ is observed at 298 K, i.e., an abrupt drop from 92.89% (${C}_{0}=30$ mg L^−1^) to 48.85% (${C}_{0}=100$ mg L^−1^). The average value of the Hg(II) removal at 298 K is 73.93% (Fig. 4c, black markers), which confirms that the efficiency of the adsorbent material must be analyzed based on the removal percentage.

**Figure 5:**
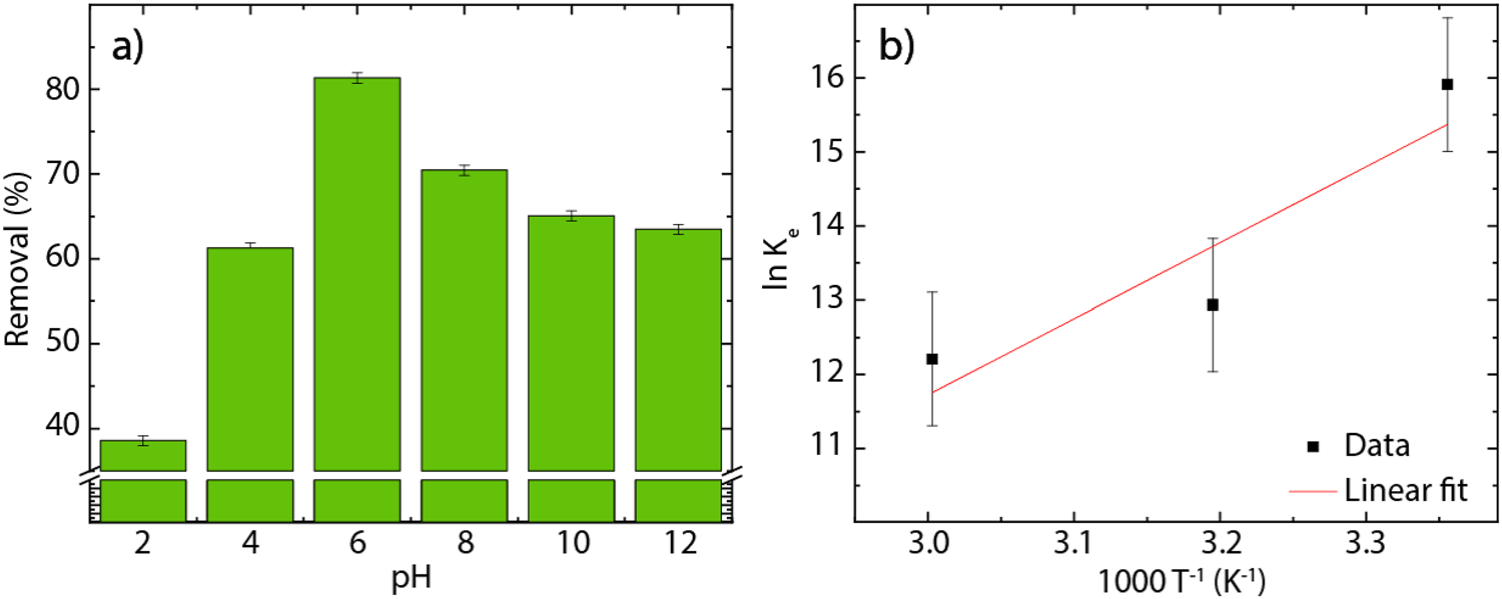
Adsorption mechanism. (**a**) Hg(II) adsorption as a function of the initial pH (Hg(II) concentration 100 mg L^−1^) and (**b**) Van’t Hoff plot for the adsorption of Hg(II) on rGO.

The effect of the initial pH on the removal of Hg(II) ions is shown in Fig. 5a. As known, HgO is not completely soluble in water at pH values above 8 since it begins to precipitate, remaining in the aqueous solution. To elucidate this fact, the experiments were carried out at six different pH values ranging from 2 to 12 and setting the temperature at 298 K. The adsorption increases starting from a removal percentage of about 39% at pH $= 2$ up to about 80% at pH $= 6$. The removal percentage remains relatively constant for $8\le$ pH $\le 12$ with an average value of 65.67%. The decrease in the removal effectiveness of Hg(II) ions at high pH values ($>8$) can be attributed precisely to the poor solubility of mercury oxide.

Regarding the adsorption process of MB as a function of pH (Fig. S7), the removal percentage of rGO is diminished for pH values less than 4 and greater than 9, and the maximum removal percentage of MB was found to be $\sim 92$% at pH = 7. These results prove that rGO could be efficiently used to remove cationic pollutants (i.e., Hg(II) and MB) at “*neutral*” pH values.

Back to Hg(II), when the pH increases from 2 to 4 the amount of Hg(II) ions adsorbed at equilibrium (${q}_{e}$) increases from 38.57 to 61.24 mg g^−1^. The decrease in the removal effectiveness of Hg(II) ions at low pH values can be attributed to competition between Hg(II) ions and H ions for the active sites of rGO. H ion is a strong competitor for adsorption due to its small size^51^. As pH increases, the repulsive forces begin to disappear and Hg(II) ions can easily interact with the negatively charged surface of rGO by electrostatic attractions. The latter statement is not entirely true, since GO rich in oxygen functional groups should report higher adsorption values (e.g., GONR in Ref.^43^). To clarify this fact, we have carried out theoretical calculations based on the density functional theory (DFT) approach considering molecular and periodic models (discussed below).

### Hg(II) sorption thermodynamics

To obtain information about the energy changes due to the involved adsorption process, the Gibbs free energy (${\Delta G}^{0}$), enthalpy change (${\Delta H}^{0}$), and entropy change (${\Delta S}^{0}$) were estimated following the approach found in Refs.^52–54^:

9 $${K}_{e} =\frac{{K}_{L} \cdot {M}_{A}}{{\gamma }_{e}}$$

10 $$\mathrm{ln} \frac{{K}_{e}}{{\gamma }_{e}}=\frac{{\Delta S}^{0}}{R}-\frac{{\Delta H}^{0}}{RT}\cong \mathrm{ln}{K}_{e}$$

11 $${\Delta G}^{0}=-RT\mathrm{ln}{K}_{e}$$

where ${K}_{L}$ is the Langmuir constant, ${K}_{e}$ is the equilibrium constant (unit less), ${\gamma }_{e}$ is the activity coefficient, and ${M}_{A}$ represents the molar weight of Hg^55^. $T$ is the absolute temperature and $R$ is the gas constant. To apply the above equations, the activity coefficient of the sorbent should be estimated from Debye–Huckel limiting law, or infinite dilute value of the equilibrium constant (${K}_{e}$) as $\gamma \cong 1$^53^. The latter is used in the present work (Table 2).

**Table 2:** Thermodynamics parameters for Hg(II) adsorption on rGO at different temperatures.

T (K)	${K}_{e}$	$\Delta {G}^{0}$(kJ mol^−1^)	$\Delta {H}^{0}$(kJ mol^−1^)	$\Delta {S}^{0}$(kJ mol^−1^ K^−1^)
298	$8.14\cdot {10}^{6}$	− 39.43		
313	$0.31 \cdot {10}^{6}$	− 32.93	− 98.31	0.085
333	$0.12 \cdot {10}^{6}$	− 32.30		

The values of ${\Delta H}^{0}$ and ${\Delta S}^{0}$ were calculated from the slope and intercept of Van’t Hoff plot of $\mathrm{ln} {K}_{e}$ as a function of ${T}^{-1}$. The Van’t Hoff plot and estimated parameters are shown in Fig. 5b and Table 2, respectively.

The negative $\Delta {G}^{0}$ values observed at different temperatures indicate spontaneous adsorption of Hg(II) ions onto the rGO surface. It is worth noting that for $\Delta {G}^{0}$ values in the range from 0 to − 20 kJ mol^−1^, the adsorption process is assigned to physisorption (multilayer adsorption), while in the range from − 80 to − 400 kJ mol^−1^, the adsorption is assigned to chemisorption (monolayer adsorption)^36^. The partition between these two ranges is ambiguous. Then the estimated $\Delta {G}^{0}$ values in the range from − 39.43 to − 32.30 kJ mol^−1^ (Table 2) suggest that the adsorption process of Hg(II) ions on rGO is governed by a mixed physisorption-chemisorption process as observed for MB^36^. As the temperature increases, the $\Delta {G}^{0}$ value decreases by 16% at 313 K and by 18% at 333 K. The negative $\Delta {H}^{0}$ value of − 98.31 kJ mol^−1^ infers an exothermic nature, advising a negative impact on the adsorption of Hg(II) ions. The positive value of $\Delta {S}^{0}=$ 0.085 kJ mol^−1^ K^−1^ implies the affinity of Hg(II) ions toward the rGO surface.

### DFT calculations

**Figure 6:**
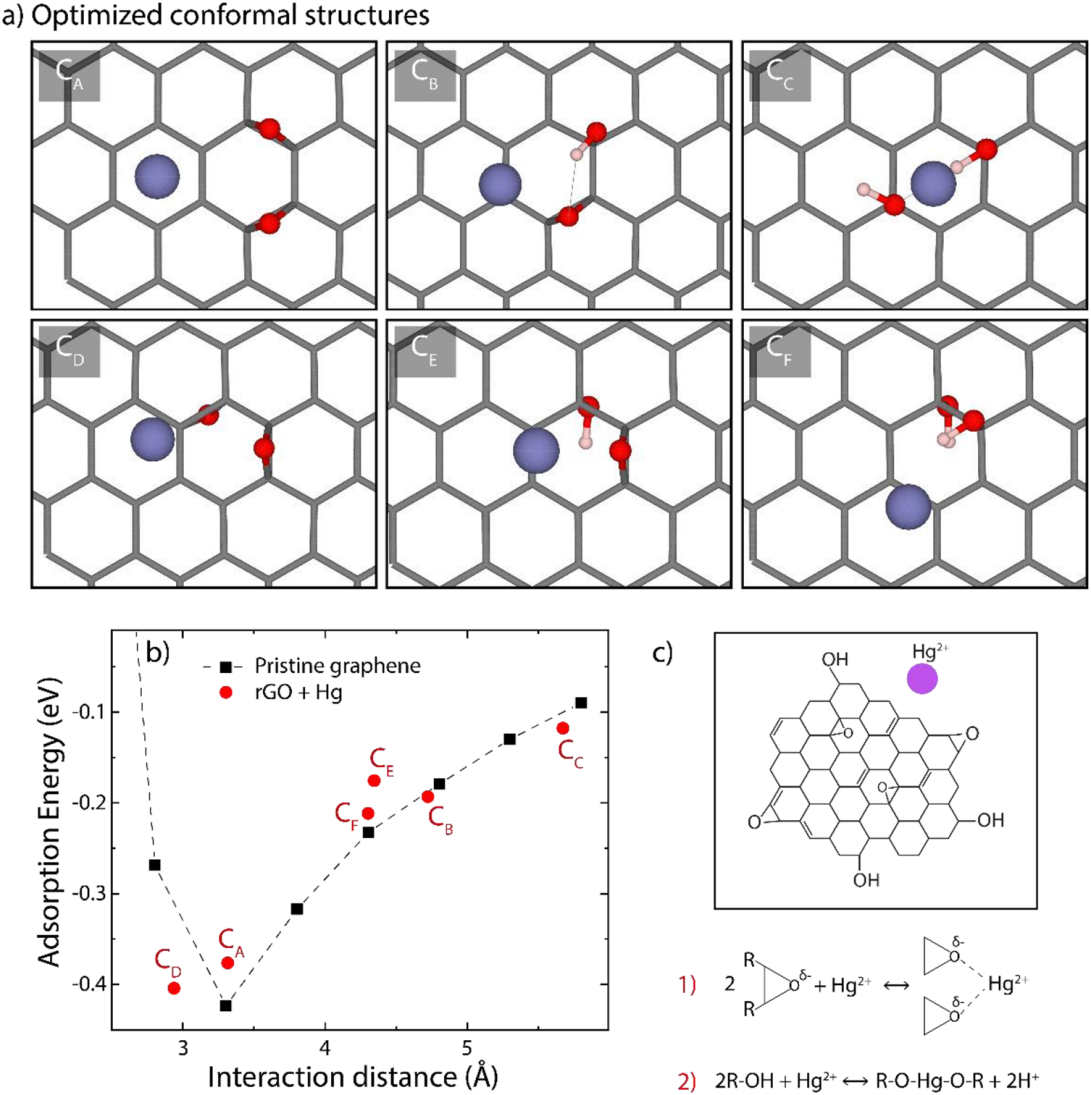
DFT computations. (**a**) Optimized rGO structures interacting with a single Hg atom, (**b**) Adsorption energy and interaction distance of the Hg atom as a function of the position of the oxygen-functional groups on the rGO surface, and (**c**) the proposed chemical reaction mechanism: 1 represents physisorption and 2 represents chemisorption. (**a**) was produced using the VMD software^56^, version 1.9.3, available at: https://www.ks.uiuc.edu/Research/vmd/.

The calculations were performed at the DFT level by means of the Gaussian16 and VASP codes. All structures were optimized without any symmetry restriction. It must be pointed out that, although the structure and chemical composition of rGO remains unclear so far, it is widely accepted that epoxy (–C–O–C–) and hydroxyl (–C–OH) are the dominant functional groups, and these oxygen-containing functional groups are distributed on the rGO surface randomly. Therefore, in setting up the theoretical adsorption study, we have considered six different configurations of functionalized graphene. Each model contains 72 carbon atoms and two O-containing groups. In Fig. S8 we report a graphical representation of the six rGO models (C_A_,…C_F_) in their equilibrium configurations before the Hg adsorption. The theoretical adsorption is then studied by placing a Hg atom per unit cell in the vicinity of the rGO model and allowing the system to relax to the equilibrium configuration. The equilibrium configurations for Hg adsorption on each model are shown in Fig. 6a.

The adsorption energy (${E}_{ads}$) is estimated as follows:

12 $${E}_{ads}={E}_{T(surf+Hg)}-\left({E}_{T\left(surf\right)}+{E}_{T(Hg)}\right)$$

where ${E}_{T(surf+Hg)}$, ${E}_{T\left(surf\right)}$, ${E}_{T(Hg)}$ represent the total energy of rGO + Hg system, rGO structure, and single Hg atom, respectively.

We find negative adsorption energies in all cases, which means that the adsorption of Hg is energetically favored (Tables S4 and S5). A detailed analysis of the adsorption energy shows that the most favorable configurations for Hg adsorption are the C_A_ (${E}_{ads}=-0.38$ eV) and C_D_ (${E}_{ads}=-0.40$ eV) with a Hg-surface distance of 3.32 $\text{\AA}$ and 2.94 $\text{\AA}$, respectively (Fig. 6b). Other configurations, with the Hg atom adsorbed further away from the rGO plane, are also found, suggesting that the main interaction mechanism has a leading dispersive nature. Additional structural parameters (DHg-O or DHg-C), adsorption energies, Bader charges are reported in Tables S4–S6.

In all configurations, the Hg atom is usually found relatively far away from the O-containing groups. This result suggests that Hg(II) ions seek oxygen-free zones for their adsorption, instead of what was previously supposed, i.e., only oxygen-containing functional groups favor the adsorption process through electrostatic attractions. The latter confirms our statement that the adsorption process of Hg(II) on rGO includes physisorption and chemisorption with a possible chemical reaction mechanism depicted in Fig. 6c.

To further elucidate this fact, we have evaluated the adsorption energy of Hg on pristine graphene at different distances by locating the Hg atom in the center of a hexagon (Fig. 6b, dashed black line). Interestingly, all configurations of the Hg + rGO system follow the same trend as Hg onto graphene surface, and the minimum adsorption energy on intrinsic graphene was found to be ${E}_{ads}=-0.42$ eV at a distance of 3.30 $\text{\AA}$.

A big drawback of the periodic approach outlined so far is its impossibility to properly introduce the charge. For this reason, we have carried out an additional gas-phase adsorption study using Gaussian16. We adopted the same starting geometries as described above, saturating the dangling bonds of the frontier atoms with H atoms. These gas-phase calculations allowed us to introduce explicitly a + 2 charge in the system. In Table S5 we report the adsorption energies as obtained in the charged gas-phase study. We still find that the Hg(II) adsorption is energetically favorable. Moreover, in both cases, the adsorption energies follow the same trend. We finally point out that the adsorption energies (Table S5) for the configurations C_A_ (${E}_{ads}=-36.31$ kJ mol^−1^) and C_D_ (${E}_{ads}=-39.07$ kJ mol^−1^) match perfectly with the values estimated by the Gibbs free energy, particularly, at 298 K = $- 39.84$ kJ mol^−1^. No dramatic changes in the density of states were observed after the Hg adsorption on the most stable rGO surfaces (Fig. S9).

## Discussions

To evaluate the material obtained for the Hg(II) removal from aqueous solutions, the saturation uptake capacity (maximum adsorption capacity) and removal percentage have been deemed as two principal criteria, and high values for both of them are needed to achieve high effectiveness and efficiency for the capture of Hg(II) ions. Interesting Hg(II) saturation uptake capacity and decent removal percentage for Hg(II) have both been demonstrated in our green-prepared, partially reduced, and non-extra functionalized rGO as reported herein, which sets a novel benchmark for adsorbent graphene-based materials.

While the selectivity against a series of trace metal ions in practical applications of decontaminating Hg(II) has been not studied here, this issue is widely addressed by functionalizing rGO with thiol groups since the presence of sulfur extraordinarily improves the uptake capacity and selectivity of adsorbent material as reported in Ref.^22^ and Ref.^41^; we will investigate this aspect in the near future. We point out that the main goal of the present work is to understand the interaction between Hg(II) and as-made rGO (never reported in such detail) in order to promote new processes for the potential scalability and application of rGO in water treatment technologies.

The issues of structure stability, mainly, under different temperatures and decreasing Hg(II) affinity over a broad range of pH represent some barriers for most Hg(II) adsorbent materials; these issues have also been addressed in as-made rGO. Concerning reusability, we suggest the following well-known techniques or processes: (i) the adsorbed Hg(II)-rGO system can be separated from aqueous media by filtration using filters with a pore size less than 1 µm since rGO is within the order of a few micrometers (Fig. S1c), (ii) Hg(II) can be released from rGO by applying the concept of ionic force, i.e., by applying buffer solutions, and (iii) the isolated Hg(II) ions can be extracted by sulfide precipitation for possible commercialization. Then rGO could be used again, however, its adsorption capacity could be diminished which is under study considering different pollutants (Ca(II), Zn(II), Mg(II), Na(I)). The latter idea motivates a new extended work.

From the theoretical perspective, previously, it could be thought that oxygen functional groups, by creating a negative charge on the graphene surface, directly influenced the adsorption process of cationic contaminants, i.e., through simple Coulomb attractions. Although this is shown in the proposed mechanism 1 and 2, the DFT calculations further show an important fact, the adsorption of the Hg atom is highly influenced by the perpendicular $\pi$ electron found in the oxygen-free zones. More insight into this matter can be obtained by analyzing the adsorption of Hg(II) on GO (Fig. S4), which is rich in oxygenated functional groups but shows low removal capacity as compared to rGO (Fig. 2a). We plan to investigate this matter in the near future.

## Conclusions

In summary, we have demonstrated that our green-prepared (and partially reduced) rGO can be used for the effective and efficient removal of Hg(II) ions from aqueous solutions. rGO exhibits a good affinity for Hg(II) with fast adsorption kinetics of 20 min and saturation Hg(II) capacity of 110.21 mg g^−1^ at 298 K. These results are superior to those recently reported for other graphene-based benchmark materials.

Using several chemical physics analyses, we have also shown that our as-made rGO keeps a good efficiency over a wide range of initial Hg(II) concentrations and a broad range of pH ($>4$). Our results suggest that the rGO-Hg adsorption interaction follows a mixed physisorption-chemisorption process as also evidenced by DFT computations.

Finally, both the experiments and the theory suggest that the Hg(II) ions prefer oxygenated-free zones on the rGO surface. The present study proposes non-extra functionalized rGO as a potential green-prepared adsorbent to treat water or wastewater.

## Methods

### Materials and measurements

All chemicals were purchased in high purity and used as received without further purification. TEM analysis was performed on a JEOL JEM-1400 Plus. The scanning electron microscope analysis was performed on a JEOL JSM-IT100 IntouchScope. EDS measurements were carried out on a JEOL-made dispersive X-ray spectrometer. Absorption spectra were recorded using a Thermo Scientific Evolution 200 UV–Visible spectrophotometer. IR spectra were recorded on a Jasco FT/IR-4000 spectrometer. Raman measurements were performed on a Jasco NRS-500 spectrometer with a 532 nm laser excitation. TGA was analyzed by using a Perkin-Elmer STA-600 thermal analyzer. X-ray diffraction measurements were done using an X-ray PANalytical Pro diffractometer in the diffraction angle (2$\theta$) window of $5^\circ$ to $90^\circ$ using Cu K*α* irradiation. AAS analysis was performed on an Atomic Absorption iCE 3000 series spectrometer (iCE-3000-AA-VP100) analyzer (Method 3112-B. Determination of Hg in water by AAS-cold vapor).

### Synthesis of GO and rGO

A round-bottom flask was charged with graphite powder (1.5 g), H_2_SO_4_ (35 ml), and KMnO4 (4.5 g) under stirring in an ice-water bath. The resulting mixture was agitated by adding 75 ml distilled water at $\sim 90$ $^\circ{\rm C}$. Additionally, 250 ml distilled water were added, followed by 7.5 ml H_2_O_2_. The resulting solution was collected, washed by centrifugation with HCl solution and distilled water several times up to adjust the pH $\sim 6$, and then dried under vacuum at $80$ $^\circ{\rm C}$ for 2 h to obtain graphite oxide powder. In a typical experiment, 50 mg of graphite oxide powder was dispersed in 500 ml distilled water by sonication for 0.5 h. The resulting solution was centrifuged to separate GO from non-exfoliated graphite oxide particles. Under agitation, 1.0 g citric acid were added to the centrifuged suspension. The precipitated material was collected, washed with distilled water by centrifugation, and dried under vacuum at $80$ $^\circ{\rm C}$ for 2 h to obtain (partially-reduced) rGO powder. GO elemental analysis: C: 49.7%; O: 50.3%. rGO elemental analysis: C: 62.9%; O: 37.1%.

### Hg(II) sorption kinetics

A 300 ml aqueous of HgO (150 mg L^−1^, HCl and NaOH 0.1 N were used to adjust the pH of the solutions, pH = 6.4) was added to a falcon tube. Then 200.0 mg rGO sample was added to form a slurry. The mixture was stirred at room temperature for 8 h. During the stirring period, the mixture was filtered at intervals through a 0.45-mm membrane filter for all samples, then the filtrates were analyzed by using AAS-cold vapor to determine the remaining Hg(II) content (standard methods 3112-B; 3111-B.4b). A final remark, as the present work is part of a national project, the pH value was adjusted to 6.4, seeking to emulate the typical conditions of water contaminated by the mining industry, where it has been measured that the pH ranges between 6.2 and 6.5. Most importantly, with this pH value, the solubility of HgO in water is guaranteed.

### Hg(II) sorption isotherm

rGO powder (2.5 mg) was added to each falcon tube containing HgO solution (50 ml) with different concentrations (from 100 to 1000 p.p.b.) at 298 K, 313 K, and 333 K. The mixtures were stirred at room temperature for 0.5 h, and then were filtered separately through a 0.45-mm membrane filter, and the filtrates were analyzed by using AAS-cold vapor to determine the remaining Hg(II) content (standard methods 3112-B; 3111-B.4b).

### Periodic DFT calculations

All the periodic DFT calculations were carried out using the projector augmented wave method (PAW) as implemented in VASP (version5.3.3) package^57,58^. We adopted the GGA-PBE exchange–correlation functional^59^ and included the Van der Waals contribution to the inter-atomic forces using the Grimme D2 approach^60^. In all calculations, a plane wave cutoff energy of 710 eV was used. The Brillouin zone was sampled by Γ-centered ($4\times 4\times 1$) Monkhorst–Pack grids. A convergence energy criterion of 10^–5^ eV was imposed on the self-consistent cycles, whereas the geometry optimization was carried out until the maximum residual force was less than 0.01 eV/Å. The rGO surface was modeled using a periodic ($6\times 6\times 1$) graphene unit cell (with 72 C atoms) functionalized with two functional groups and a vacuum region in the out-of-plane direction of 15 $\text{\AA}$.

### Gas-phase DFT calculations

The Gaussian16 package^61^ was used to perform DFT molecular computations of the interacting Hg-atom on the rGO surface. The ω-B97XD functional was adopted together with the Pople’s split-valence 6-311G(d,p) basis set^62^. The Hg atom was described by employing the LanL2DZ pseudo-potential^63^. No symmetry restriction was imposed during the optimization of the structures.

## Supplementary Information

Supplementary Information.
