# Supplementary material for: Removal of mercury(II) from aqueous solution by partially reduced graphene oxide
Source: Sci Rep. 2022 Apr 19;12:6326. doi: 10.1038/s41598-022-10259-z (PMC9018808; doi:10.1038/s41598-022-10259-z)
Supplement: Supplementary file 1 — Supplementary Information. [file 41598_2022_10259_MOESM1_ESM.docx]

**Supporting Material:** **Removal of mercury(II) from aqueous solution by partially reduced graphene oxide**

Talia Tene ^1^, Fabian Arias Arias ^2,3^, Marco Guevara ^4^, Adriana Nuñez ^4^, Luis Villamagua ^5^, Carlos Tapia ^4^, Michele Pisarra ^6^, F. Javier Torres ^7,8^, Lorenzo S. Caputi ^4,9^ & Cristian Vacacela Gomez ^4,*^

^1^ Grupo de Investigación Ciencia y Tecnología de Materiales, Universidad Técnica Particular de Loja, 110160 Loja, Ecuador

^2^ Grupo de Energías Alternativas y Ambiente (GEA), Facultad de Ciencias, Escuela Superior Politécnica de Chimborazo, 060155 Riobamba, Ecuador

^3^ ITECA - Instituto de Tecnologías y Ciencias Avanzadas, Villarroel y Larrea, 060104 Riobamba, Ecuador

^4^ UNICARIBE Research Center, University of Calabria, I-87036 Rende (CS), Italy

^5^ Grupo de Fisicoquímica de Materiales & Departamento de Ciencias de la Computación y Telecomunicaciones, Departamento de Química, Universidad Técnica Particular de Loja, 110160 Loja, Ecuador

^6^ INFN, sezione LNF, Gruppo collegato di Cosenza, Cubo 31C, 87036 Rende (CS), Italy

^7^ Grupo de Química Computacional y Teórica (QCT-UR), Facultad de Ciencias Naturales, Universidad del Rosario, Bogotá, Colombia

^8^ Grupo de Química Computacional y Teórica (QCT-USFQ), Insituto de Simulación Computacional (ISC-USFQ), Departamento de Ingeniería Química, Universidad San Francisco de Quito, Diego de Robles y Vía Interoceánica, Quito, Ecuador

^9^ Surface Nanoscience Group, Department of Physics, University of Calabria, Via P. Bucci, Cubo 33C, I-87036 Rende, Italy

* Corresponding author: [cvacacela@yachaytech.edu.ec](mailto:cvacacela@yachaytech.edu.ec), [fernandoj.torres@urosario.edu.co](mailto:fernandoj.torres@urosario.edu.co)

**Materials:**

Graphite powder (<150 $\mu$m, 99.99%), sulfuric acid (H_2_SO_4_, ACS reagent, 95.0–98.0%), potassium permanganate (KMnO_4_, ACS reagent, $\geq$99.0%), hydrochloric acid (HCl, ACS reagent, 37%), and citric acid (C_6_H_8_O_7_, ACS reagent, $\geq$99.5%) were obtained from Sigma Aldrich. All chemicals were used as received, without further purification.

**Samples preparation**:

SEM samples were prepared by drop casting on aluminum substrates and dried at 80 $℃$ for 2 h. Similarly, TEM and Raman samples were prepared by drop casting onto formvar-coated copper grids and glass substrates, respectively.

**Supplementary Figures: Synthesis and characterization of GO and rGO**


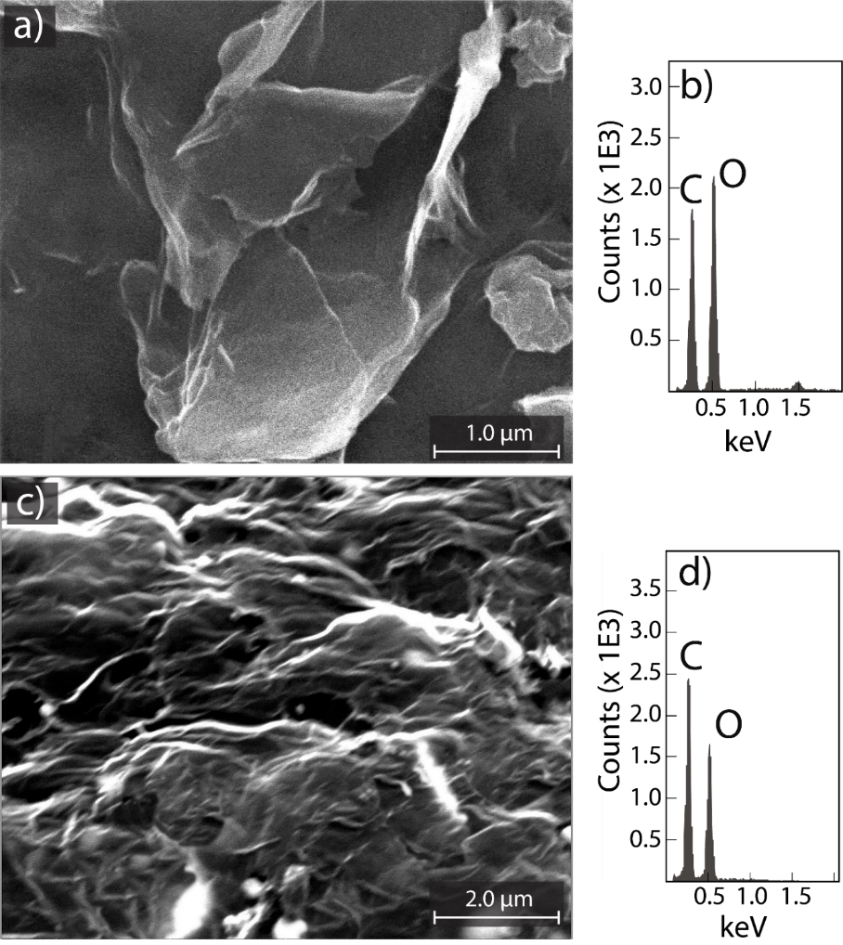


**Figure S1.** SEM morphology and EDS analysis of (a)-(b) GO, and (c)-(d) rGO.


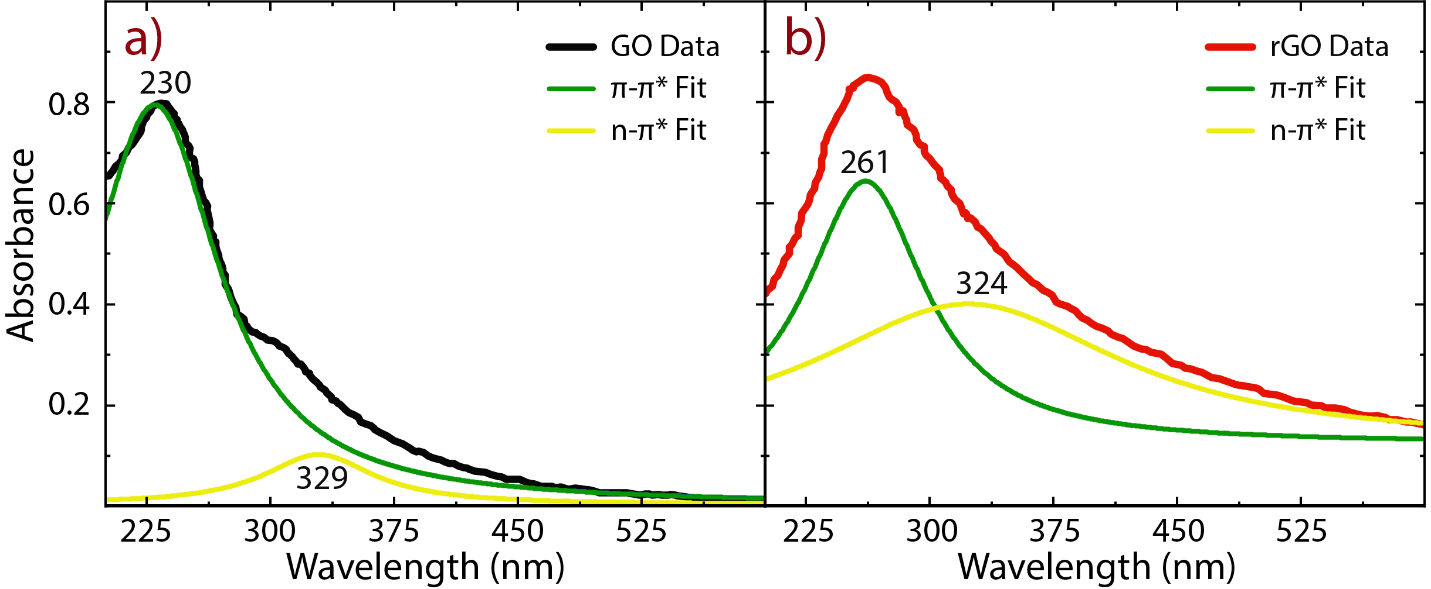


**Figure S2.** UV-Vis spectra of (a) GO and (b) rGO. To scrutinize the transformation of GO into rGO, the absorbance spectra were fitted with two Lorentzian functions. Adapted from Ref. 36.


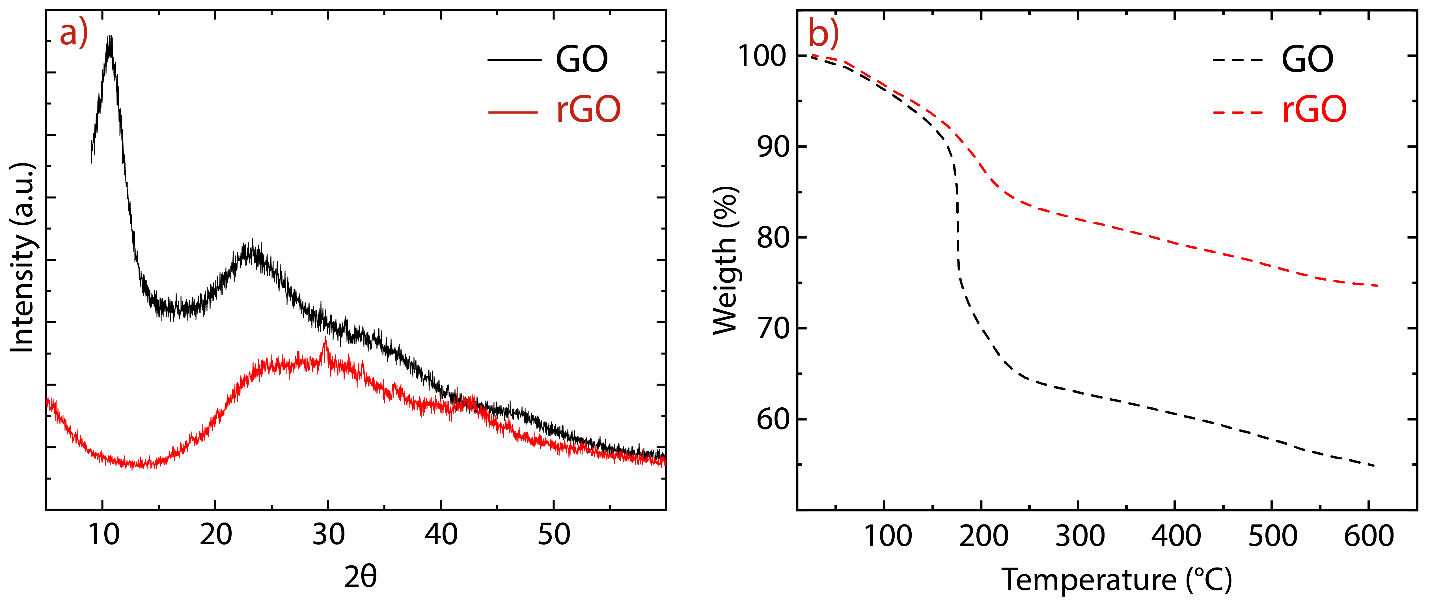


**Figure S3.** (a) XRD patterns of GO and rGO and (b) TGA analysis of GO and rGO.

**Supplementary Figures: Methyl Blue (MB) sorption on rGO**


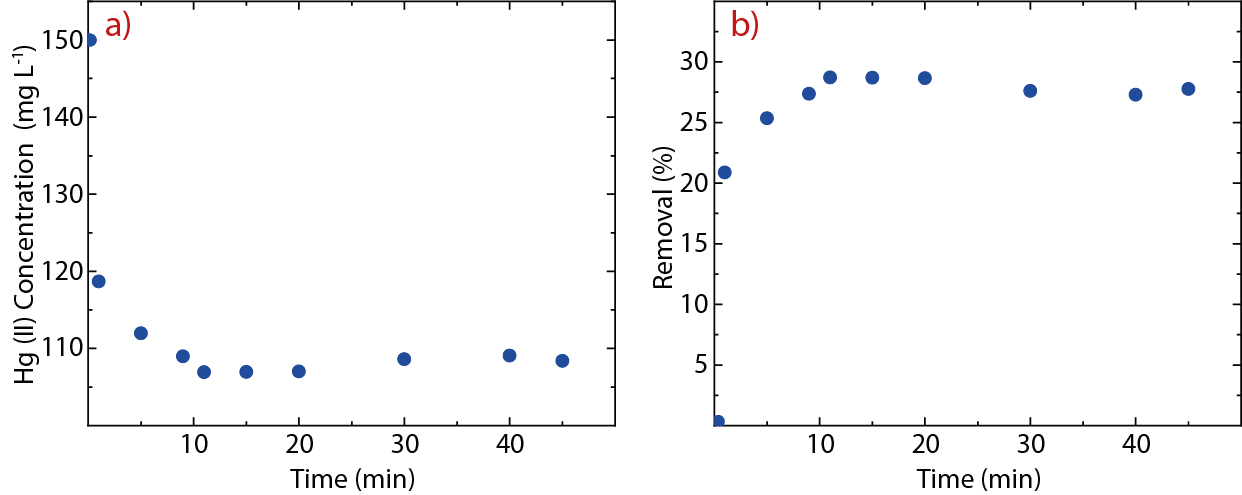


**Figure S4.** (a) Hg(II) adsorption kinetics of GO under the Hg(II) initial concentration of 150 mg L^-1^. (b) The corresponding removal percentage.


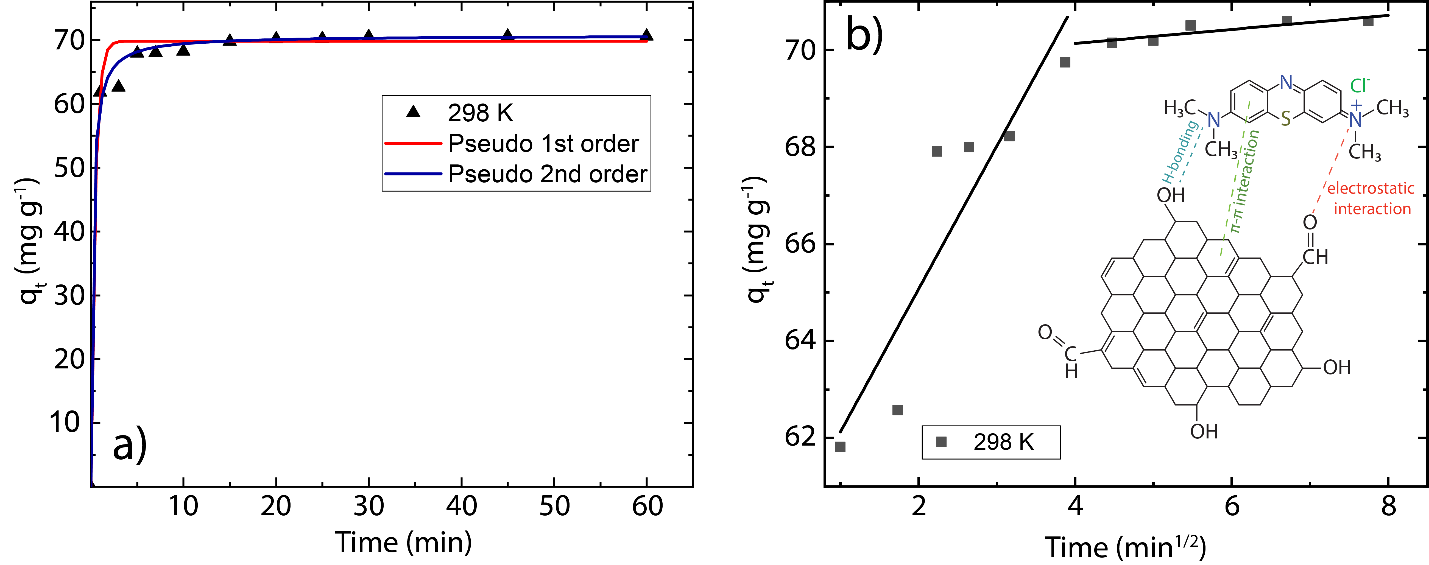


**Figure S5.** (a) Adsorption kinetics of MB on rGO as a function of contact time (up to 60 min) at 298 K and (b) Intraparticle diffusion (IPD) plot showing two regions of linearity. Adapted from Ref. 36.


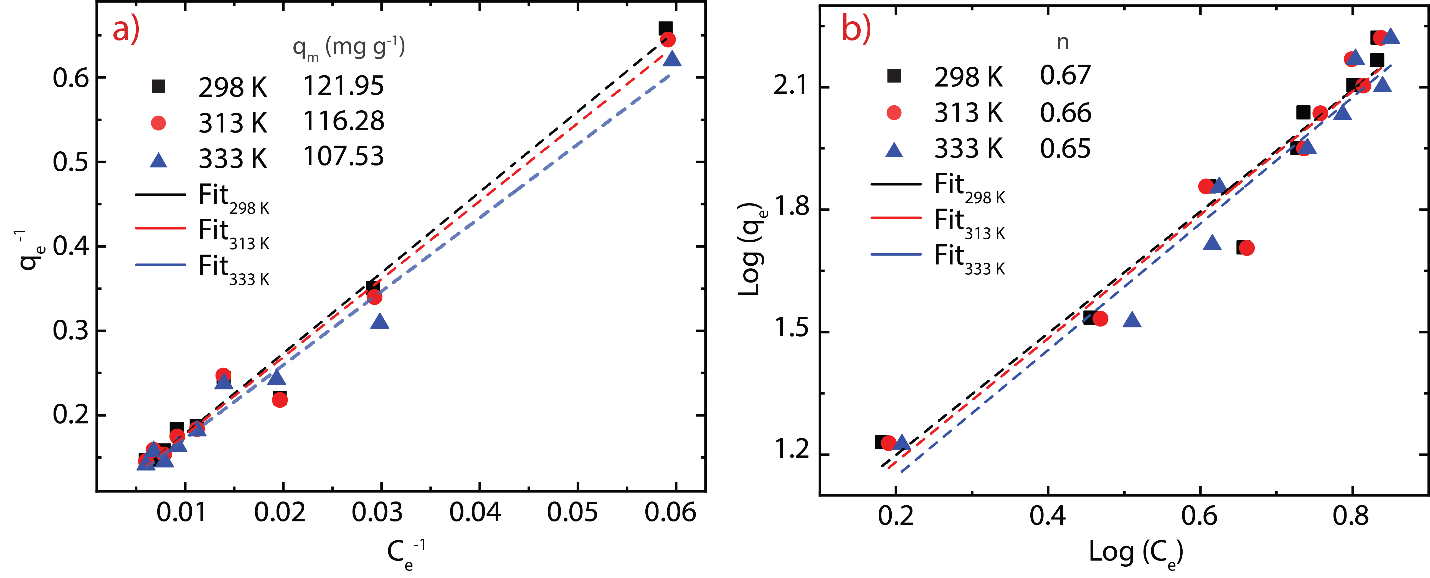


**Figure S6.** Adsorption isotherms of MB on rGO at three different temperatures. (a) Langmuir model and (b) Freundlich model. Adapted from Ref. 36.


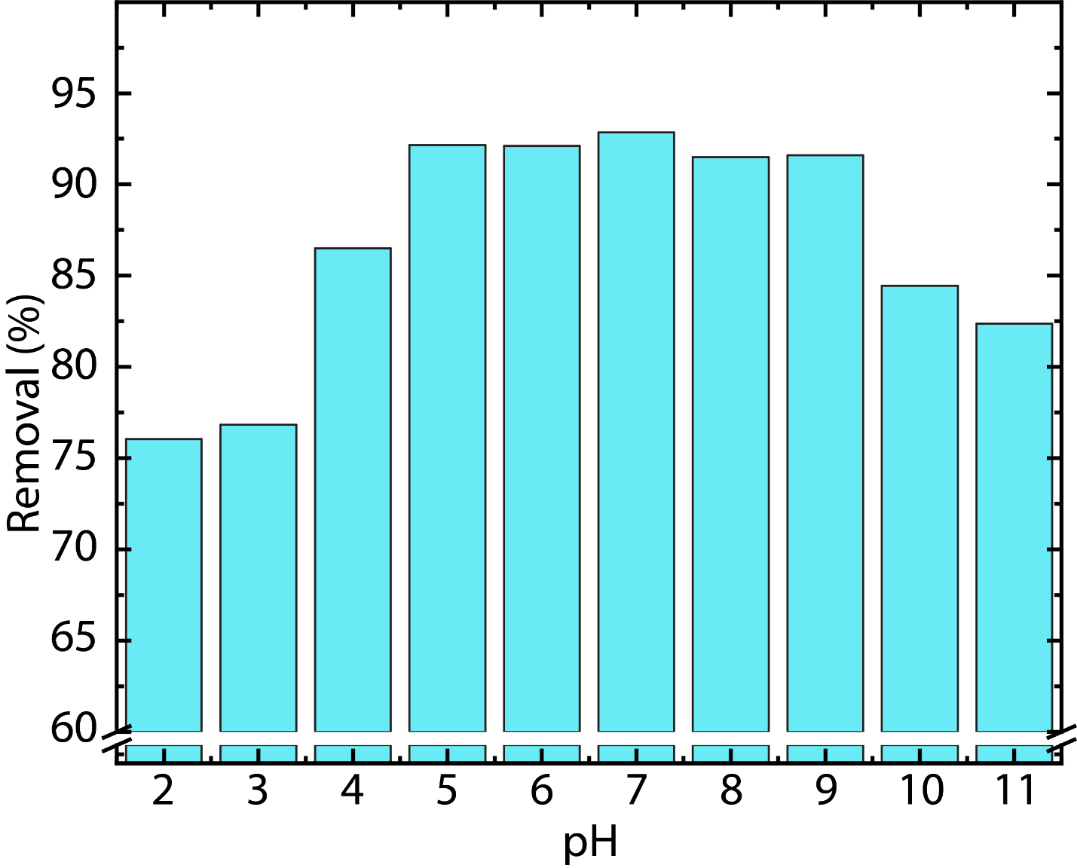


**Figure S7.** Adsorption of MB on rGO as function of the pH. Adapted from Ref. 36.

**Supplementary Figures: DFT study**


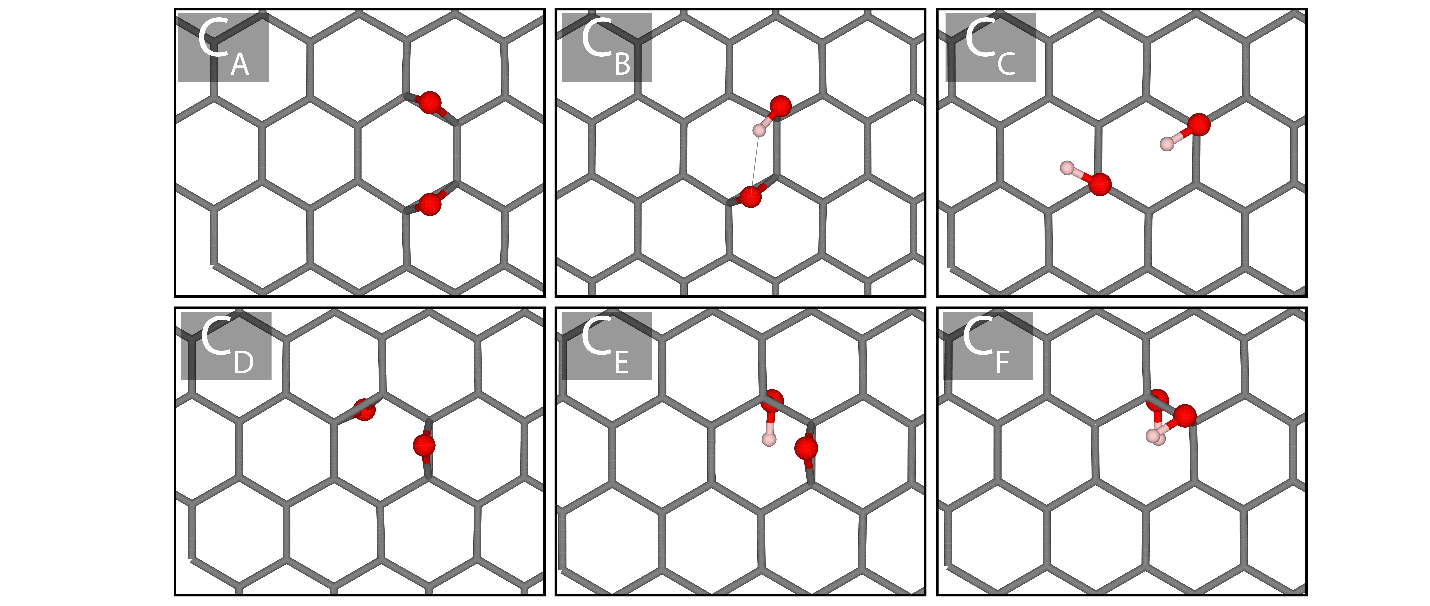


**Figure S8.** Optimized pristine rGO structures. Figure 6a was produced using the VMD software [see Ref. 56 in the main text], version 1.9.3, available at: https://www.ks.uiuc.edu/Research/vmd/


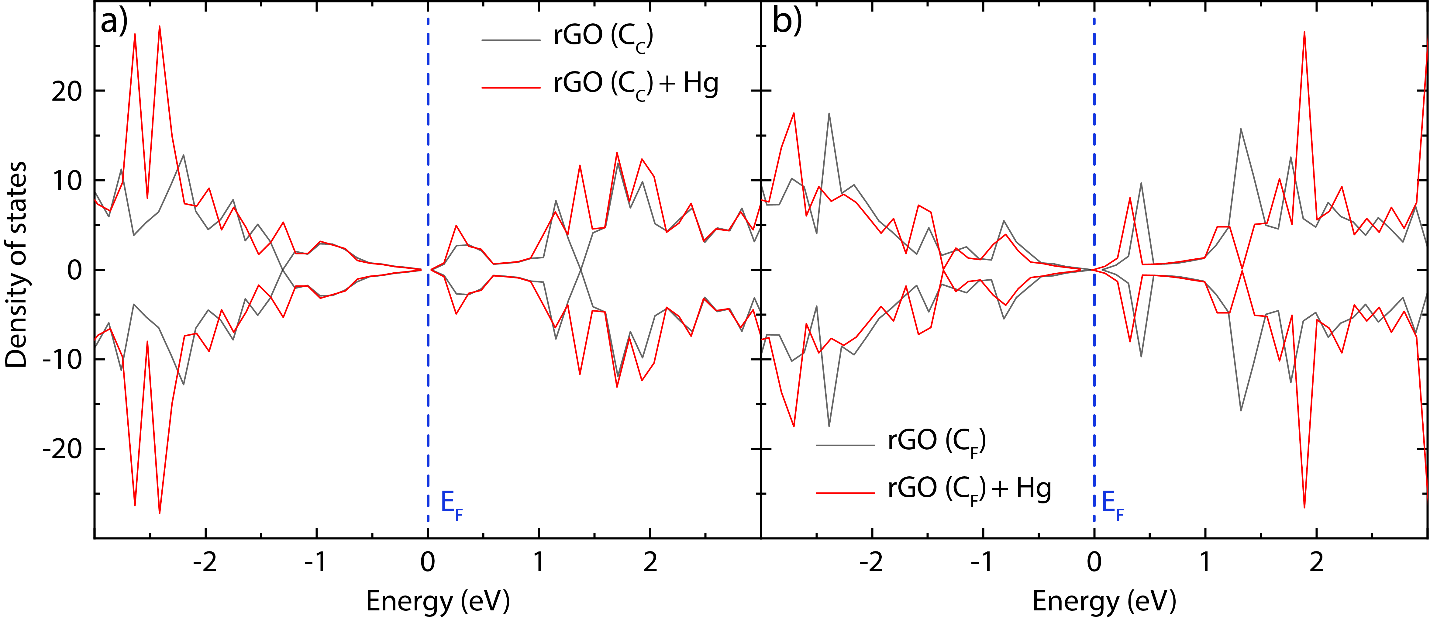


**Figure S9.** Density of states (DOS) of the most stable rGO structures: pristine rGO (black line) and rGO + Hg (red line).

**Supplementary Tables:**

**Table S1.** Parameters of the pseudo-first-order and pseudo-second-order kinetic model at 298 K.

|  | | **Temperature** |
| --- | --- | --- |
| **Parameters** | | **298 K** |
| q_e(exp)_ (mg g^-1^) | | 142.26 |
| **Pseudo-first-order model** | |  |
| q_e(cal)_ (mg g^-1^) |  | 143.7 ± 5.7 |
| k_1_ (min^-1^) |  | 0.194 ± 0.030 |
| SSE |  | 1826 |
| R^2^ |  | 0.949 |
| RMSE |  | 8.546 |
| **Pseudo-second-order model** | |  |
| q_e(cal)_ (mg g^-1^) |  | 151.3 ± 9.45 |
| k_2_ (g mg^-1^ min^-1^) |  | 0.002± 0.001 |
| SSE |  | 2480 |
| R^2^ |  | 0.931 |
| RMSE |  | 9.96 |

**Table S2.** Parameters of the intraparticle diffusion (IPD) model for the Hg(II) adsorption on rGO at 298 K

| **Parameters** | **Value** |
| --- | --- |
| K_p_ (mg g^-1^ min^-1/2^) | 7.82 ± 1.25 |
| C (mg g^-1^) | 44.28 ± 7.75 |
| R_i_ | 0.491 |
| R^2^ | 0.963 |

**Table S3.** Parameters of Langmuir and Freundlich isotherm models, considering three different temperatures.

| **T (K)** | **Langmuir model** | | | **Freundlich model** | | |
| --- | --- | --- | --- | --- | --- | --- |
|  | **K_L_ (L g^-1^)** | **q_m(cal)_ (mg g^-1^)** | **R^2^** | **K_F_ (mg^(1-n)^ g^-1^ L^1/n^)** | **n** | **R^2^** |
| 298 | 4.71 | 110.21 | 0.933 | 0.592 | 1.44 | 0.936 |
| 313 | 1.56 | 217.34 | 0.947 | 0.872 | 0.57 | 0.979 |
| 333 | 0.58 | 255.04 | 0.964 | 0.885 | 0.26 | 0.978 |

**Table S4.** Calculated parameters: adsorption energy, Bader charge transfer, and interaction distance of rGO + Hg system.

| **rGO Surface** | **Adsorption energy (eV)** | **Bader charge (e)** | **DHg-O (****Å)** | **DHg-C (Å)** |
| --- | --- | --- | --- | --- |
| C_A_ + Hg | -0.38 | 0.001 | 3.65 | 3.32 |
| C_B_ + Hg | -0.19 | 0.015 | 3.62 | 4.72 |
| C_C_ + Hg | -0.12 | 0.010 | 3.80 | 5.67 |
| C_D_ + Hg | -0.41 | 0.009 | 3.42 | 2.94 |
| C_E_ + Hg | -0.18 | 0.013 | 3.58 | 4.35 |
| C_F_ + Hg | -0.21 | 0.018 | 3.81 | 4.30 |

**Table S5.** Adsorption energy predicted by VASP and Gaussian 09 for the different rGO + Hg systems under study (Fig. 6a)

| **Adsorption Energy (kJ mol^-1^)** | | |
| --- | --- | --- |
| **System** | **VASP** | **GAUSSIAN 09** |
| C_A_ + Hg | -36.31 | -25.88 |
| C_B_ + Hg | -18.66 | -16.60 |
| C_C_ + Hg | -11.37 | -12.51 |
| C_D_ + Hg | -39.07 | -27.54 |
| C_E_ + Hg | -16.94 | -19.79 |
| C_F_ + Hg | -20.42 | -19.85 |

**Table S6.** Bader charge calculations of oxygenated functionals groups

| **rGO surface** | **Bader charge (e)** | | |
| --- | --- | --- | --- |
|  | **Atom** | **Before adsorption** | **After adsorption** |
| C_A_ | O_a_ | -0.797 | -0.793 |
|  | O_b_ | -0.799 | -0.794 |
| C_B_ | O_a_ | -0.814 | -0.814 |
|  | O_b_ | -1.017 | -1.005 |
|  | H_a_ | 0.591 | 0.575 |
| C_C_ | O_a_ | -1.016 | -1.020 |
|  | O_b_ | -1.028 | -1.051 |
|  | H_a_ | 0.589 | 0.612 |
|  | H_b_ | 0.570 | 0.569 |
| C_D_ | O_a_ | -0.780 | -0.781 |
|  | O_b_ | -0.780 | -0.781 |
| C_E_ | O_a_ | -0.792 | -0.011 |
|  | O_b_ | -1.008 | 0.006 |
|  | H_a_ | 0.551 | -0.002 |
| C_F_ | O_a_ | -1,024 | -1,036 |
|  | O_b_ | -1,020 | -1,035 |
|  | H_a_ | 0,560 | 0,571 |
|  | H_b_ | 0,563 | 0,566 |
